# Double and single mixed-lineage leukemia-rearranged subclones in pediatric acute myeloid leukemia: a case report

**DOI:** 10.1186/s13256-021-02841-2

**Published:** 2021-05-07

**Authors:** Mary McGrath, Gayle Smink

**Affiliations:** 1 Division of Pediatric Hematology and Oncology, Department of Pediatrics, University of Michigan, Ann Arbor, MI USA; 2 Division of Pediatric Hematology and Oncology, Department of Pediatrics, Penn State College of Medicine and Penn State Children’s Hospital, 500 University Drive, Mail Code H085, Hershey, PA USA

**Keywords:** Leukemia, Acute myeloid leukemia, Mixed-lineage leukemia

## Abstract

**Background:**

Acute myeloid leukemia (AML) is a disease with a significant amount of cytogenetic heterogeneity including mixed-lineage leukemia (MLL) gene rearrangements. Pediatric AML commonly has genetic rearrangements which involve chromosome 11q23 in 15–20% of cases, and these genetic abnormalities have been associated with a poorer prognosis (Grimwade *et al.* in Blood 92:2322–2333, 1998; Raimondi *et al.* in Blood 94:3707–3716, 1999; Lie *et al.* in Br J Haematol 122: 217–225). MLL rearrangements in AML have been shown to have multiple different fusion partners (Meyer *et al.* in Leukemia 23:1490–1499). Heterogeneity of these cytogenetic abnormalities makes it difficult to determine how to approach patients from a treatment standpoint. This difficulty is further complicated when patients have more than a single MLL rearrangement.

**Case presentation:**

A 10-year-old Caucasian girl presented with easy bruising and was found to have acute myeloid leukemia. Her cytogenetics showed two different MLL rearrangements, t(9;11)(p22;q23) and t(11;19)(q23;p13.3). At initial presentation she had no other cytogenetic findings. She responded well to initial therapy and achieved remission following the first induction cycle and completed four rounds of chemotherapy. She subsequently had a relapse of her AML, and her cytogenetics were consistent with a single MLL rearrangement, t(9;11)(p22;q23), in addition to monosomy 7. She was treated with reduction therapy and a haplo-identical bone marrow transplant but ultimately succumbed to her disease.

**Conclusion:**

MLL rearrangements are common in AML, but clinical significance continues to be elusive, and there is conflicting data on the prognostic significance. In the setting of multiple MLL rearrangements, there is concern for reduced survival, although treatment modifications are not currently done in this setting. This report details a case with multiple MLL rearrangements that initially responded to therapy but ultimately had disease progression with a selection of a leukemic clone containing a single MLL rearrangement.

## Background

When considering treatment of acute myeloid leukemia (AML), response to induction therapy and specific cytogenetic abnormalities such as t(8;21), inv(16), NPM, CEBPα, FLT3/ITD+, monosomy 7, monosomy 5, and del5q are the mainstays used to determine risk-directed therapy. The impact on prognosis is known for some cytogenetic abnormalities such as t(8;21), inv(16), and t(15;17), but other abnormalities are rare and their significance difficult to ascertain [1]. There are numerous other common cytogenetic findings that are not routinely used for risk stratification, including 11q23 rearrangements, despite their prevalence in pediatric AML. Mixed-lineage leukemia (MLL) rearrangements are found in 15–20% of pediatric AML cases [2]. In general, patients with MLL rearrangements have poorer outcomes compared to non-MLL-rearranged leukemia [2–4]. There are many defined fusion partners of MLL, although the most common seen in AML is the t(9;11)(p21;q23) fusion [5]. MLL-AF6, MLL-AF9, MLL-ENLs, MLL-AF10, and the ALL-AF17 fusion proteins are the most commonly encountered translocations in AML [6]. The significance of multiple MLL rearrangements is unknown, but the concern is that this may lead to reduced survival. This case report details a patient with two MLL rearrangements who progressed to more aggressive disease with emergence of a single MLL rearrangement along with monosomy 7.

## Case presentation

The patient is a 10-year-old Caucasian girl who presented to her pediatrician with a 2-week history of excessive bruising. At presentation she had no significant family history of childhood illnesses or malignancies. On physical exam she had significant bruising and pallor, but without any other exam findings. An initial complete blood count (CBC) revealed leukocytosis (white blood cell count 36.3k/µL), anemia (hemoglobin 9.1 g/dL), thrombocytopenia (platelets 31k/µL), and circulating blasts (absolute blast count 22.76k/µL). Peripheral blood flow cytometry was consistent with AML. She had no central nervous system (CNS) involvement. Chromosomal findings at diagnosis were 46,XX,t(11;19)(q23.2;p13.3)[18]/47, idem, and +6 [2]. She had a unique cytogenetic findings, which showed two different MLL rearrangements, t(9;11)(p22;q23) in 88.4% of cells and t(11;19)(q23;p13.3) in 84.4% of cells, for 18 of 20 metaphases analyzed. The interpretation stated that “interphase nuclei demonstrated an MLL gene rearrangement and a MLLT3/MLL fusion.” Fluorescence *in situ* hybridization (FISH) findings indicated that 86% of nuclei had MLL gene fusions associated with both MLLLT3/MLL(KMT2A) and MLL(KMT2A)/MLLT1 (Table 1). No other high- or low-risk abnormalities were found on cytogenetics, including negative for monosomy 7, monosomy 5, del5q, inv(16), t(8;21), FLT3/ITD, NPM1, and CEBP. The significance of this combination of MLL rearrangements was unknown, and we opted to treat per the Children’s Oncology Group AAML1031 Arm A regimen. She obtained a negative minimal residual disease (MRD) by flow cytometry following her first induction cycle and was classified as low risk, forgoing a bone marrow transplant as part of her upfront therapy. FISH studies following induction I were negative for both the t(9;11)(p22;q23) and t(11;19)(q23;p13.3) fusion. Her therapy included cytarabine, daunorubicin, and etoposide for induction I and II, cytarabine and etoposide for intensification I, and cytarabine and mitoxantrone for intensification II. Unfortunately, 5 months following the completion of therapy she had an isolated bone marrow relapse. Cytogenetic analysis of 20 metaphases revealed just a single MLL rearrangement, t(9;11)(p22;q23) (MLLT3/MLL fusion as she had at diagnosis), and in addition monosomy 7 in 19 of these metaphases. FISH confirmed that 90% of nuclei had monosomy 7 and MLL(KMT2A)/MLLT1 fusion. She had no other new cytogenetic abnormalities. Relapse therapy goals for her were to proceed in second remission to a bone marrow transplant with the best available donor and conditioning with busulfan and fludarabine. In relapse she was refractory to several re-induction therapies including TVTC (topotecan, vinorelbine, thiotepa, clofarabine) and decitabine with gemtuzumab [7, 8]. She subsequently enrolled in a phase I clinical trial after failing these re-induction regimens, and was able to achieve a low MRD and proceed to a haplo-identical stem cell transplant. Unfortunately, she was found to have florid relapse of her disease around day +100 post-transplant and passed away shortly thereafter.

**Table 1:** Fluorescence *in situ* hybridization findings at diagnosis and relapse

Abnormality name	% Abnormal at diagnosis	% Abnormal at relapse
11q23(MLL sep)	88.5	91.5
t(9;11) MLLT3/MLL fusion	88.4	
t(11;19) MLL/MLLT1 fusion	84.4	91.6
−7(D7Z1,D7S486x1)		86.5
Result	Nuc ish(MLLT3x2, MLLx3)(MLLT3 con MLLx1)[442/500]	Nuc ish(D7Z1, D7S486)x1[173/200], (MLLx3, MLLT1x2)(MLL con MLLT1x1)[458/500]

## Discussion and conclusion

The mixed-lineage leukemia gene, located on chromosome 11q23, is involved in the majority of 11q23 rearrangements seen in AML. The MLL gene plays an important role in fetal hematopoiesis, and many of the MLL gene fusion partners play a role in transcriptional regulation [9]. Amongst the various rearrangements, t(9;11)(p22;q23) and t(11;19)(q23;p13.3) are the second and third most common rearrangements involving the MLL gene in pediatric AML, respectively [9]. Although MLL gene rearrangements are seen in about 15–20% of pediatric patients with AML, there are no known cases in the literature that report two separate MLL gene rearrangements [10–12]. Our patient had both abnormalities, and some MLL gene rearrangements have been associated with poor outcomes [10–12].

The presence of these abnormalities can cause treatment dilemmas, as there are more than 50 identified MLL fusion partners, each with varying prognostic indications [8]. To complicate matters further, certain MLL rearrangements are so rare that the risk they carry in regard to relapse, event-free survival (EFS), and overall survival is yet to be determined. In addition, the prognosis associated with certain MLL rearrangements differs amongst studies. Conflicting data on prognostic significance makes it difficult to interpret these findings on an individual basis.

While the t(9;11) rearrangement is one of the more prevalent MLL rearrangements identified in AML cases, there is no consensus in the literature on its prognostic indication. Rubnitz *et al.* published that t(9;11) was a favorable prognostic marker (5-year EFS 64.9 ± 11.1%), compared to other cytogenetic markers, including several other MLL rearrangements (24.2 ± 9.4%) [13]. In particular, favorable outcomes were seen in patients with FAB-M5 AML [13]. Patients treated on the Nordic Society for Pediatric Hematology and Oncology (NOPHO) protocols likewise had favorable outcomes when a t(9;11) rearrangement was present (EFS 86 ± 9%) [12]. In the United States, on the other hand, this specific rearrangement was found to have a poor prognosis, similar to other MLL rearrangements [14]. It has been proposed that the favorable prognosis seen in the first two studies is a result of administration of cumulative high-dose cytarabine in both protocols. However, Balgobind *et al.* could not confirm the use of cumulative high-dose cytarabine leading to improved outcomes in patients with this translocation [2]. Favorable outcomes from the St. Jude Children’s Research Hospital protocols have been further attributed to the predominance of patients with FAB-M5 AML and the use of epipodophyllotoxins, which have been shown to be effective against M5 AML [13]. While this does not explain the success of the NOPHO protocols, Balgobind *et al.* also found that patients with FAB-M4 t(9;11) had much better prognosis than other FAB types [9]. This indicates that FAB type could potentially be used in risk stratification of these patients.

Von Neuhoff *et al.* found patients with t(9;11)(p22;q23) or t(11;19)(q23;p13) had equivocal outcomes to patients without MLL rearrangements, with 5-year overall survival of 51 and 58%, respectively [4]. Although they found that patients with t(9; 11) alone had equivocal outcomes, patients with t(9;11)(p22;q23) and an additional cytogenetic abnormality had a significant decline in 5-year overall survival rates at just 31% [4]. Balgobind *et al.* reported that patients with t(9;11)(p22;q23) had a 5-year EFS of 50%, and those with t(11;19)(q23.2p13.3) had a 5-year overall survival rate of 49%, which is similar to the outcomes described by von Neuhoff [2]. Similar to von Neuhoff, they found that patients with a MLL rearrangement and an additional cytogenetic aberration had worse overall survival rates compared to those with just a single MLL rearrangement [2]. Since neither group specified what additional cytogenetic abnormalities these patients had, it makes it difficult to know if the finding of two distinct MLL rearrangements in our patient impacted her outcome.

Limitations of this case report include that a single patient report is difficult to generalize to other patients with MLL rearrangements. Further case series and retrospective data would be needed to determine whether multiple MLL rearrangements have clinical significance.

There remains much ambiguity regarding the significance of certain MLL rearrangements with respect to prognosis and best treatment for patients with AML. While there have been several large studies to evaluate the role t(9;11)(p22;q23) plays on prognosis in childhood AML, results are not consistent, perhaps owing to different treatment regiments. While t(11;19)(q23;p13) is the third most common rearrangement involving the MLL gene in pediatric AML, there is very limited data on the effect this translocation has on prognosis. There is even more uncertainty when more than one MLL rearrangement is present. Given the frequency in which MLL gene rearrangements are observed in pediatric AML, it seems imperative that they be included in risk stratification for therapy selection. Since risk stratification in AML many times leads to more aggressive therapies, including consideration of stem cell transplant, the clinical significance of MLL gene rearrangements needs to be better understood to use it for risk stratification. Further research is needed to elucidate the role each MLL rearrangement plays in prognosis in order to improve treatment outcomes.

## Data Availability

Data sharing is not applicable to this article as no datasets were generated or analyzed during the current study.
